# Single-Cell Image-Based Analysis Reveals Chromatin Changes during the Acquisition of Tamoxifen Drug Resistance

**DOI:** 10.3390/life12030438

**Published:** 2022-03-17

**Authors:** Han Zhao, Li F. Lin, Joshua Hahn, Junkai Xie, Harvey F. Holman, Chongli Yuan

**Affiliations:** 1 Davidson School of Chemical Engineering, Purdue University, West Lafayette, IN 47907, USA; zhao826@purdue.edu (H.Z.); lin603@berkeley.edu (L.F.L.); joshhahn@berkeley.edu (J.H.); xie161@purdue.edu (J.X.); hholman@purdue.edu (H.F.H.); 2 Purdue University Center for Cancer Research, Purdue University, West Lafayette, IN 47906, USA

**Keywords:** epigenetics, drug resistance, breast cancer, chromatin

## Abstract

Cancer drug resistance is the leading cause of cancer related deaths. The development of drug resistance can be partially contributed to tumor heterogeneity and epigenetic plasticity. However, the detailed molecular mechanism underlying epigenetic modulated drug resistance remains elusive. In this work, we systematically analyzed epigenetic changes in tamoxifen (Tam) responsive and resistant breast cancer cell line MCF7, and adopted a data-driven approach to identify key epigenetic features distinguishing between these two cell types. Significantly, we revealed that DNA methylation and H3K9me3 marks that constitute the heterochromatin are distinctively different between Tam-resistant and -responsive cells. We then performed time-lapse imaging of 5mC and H3K9me3 features using engineered probes. After Tam treatment, we observed a slow transition of MCF7 cells from a drug-responsive to -resistant population based on DNA methylation features. A similar trend was not observed using H3K9me3 probes. Collectively, our results suggest that DNA methylation changes partake in the establishment of Tam-resistant breast cancer cell lines. Instead of global changes in the DNA methylation level, the distribution of DNA methylation features inside the nucleus can be one of the drivers that facilitates the establishment of a drug resistant phenotype in MCF7.

## 1. Introduction

Drug resistance is the leading cause of cancer treatment failure worldwide [1]. Recent statistics indicate that 90% of patients who died from cancer developed some forms of drug resistance [2,3]. Several mechanisms have been proposed in recent years regarding the molecular and cellular origin of drug resistance, including drug efflux, the inhibition of apoptosis pathways, drug target modifications, and inactivation of drug activity [4,5,6,7], where epigenetic changes confer drug resistance via altering the cellular plasticity [8,9]. The cancer stem cell (CSC) hypothesis is another popular mechanism accounting for drug resistance [10,11]. This hypothesis suggests that a tumor consists of a subset of cells with increased capacity of self-renewal and differentiation, also known as CSC [12]. After chemo and/or radiation therapy, responsive cells are depleted while CSCs survive and confer resistance [13,14]. Compared to responsive cells, CSCs have distinctive epigenetic profiles. Collectively, cellular or tumor heterogeneity, particularly in epigenetics, has been long postulated to contribute to drug resistance, calling for the use of single cell-based analysis.

Single cell tools have been developed in the past decade to pinpoint the molecular origin of tumor heterogeneity, including single-cell pharmacokinetic imaging and whole cell RNA sequencing [15,16,17,18]. Most single cell studies are genetically focused and have revealed that a secondary drug resistant population exists within tumors that are not sensitive to therapy, supporting the cancer stem cell theory [15]. Other evidence, however, is accumulating from single cell ATAC-, BS-, ChIP-, and RNA- seq that suggest that massive epigenome and chromatin structural changes exist within tumors and can confer the acquisition of resistance [19,20,21]. Specifically, changes in histone modification such as loss of H3K27me3 and deposition of H3K4me3 have been found to be associated with cancer cell plasticity [19]. In addition, whole genome methylation analysis has previously found that DNA hypermethylation contributes to the development of drug resistance in lung adenocarcinoma [20].

Although it has been increasingly recognized that the epigenome plays a significant role in the acquisition of drug resistance, we are still in the infant stage of understanding how epigenetics evolves during treatment, offers tumor cell plasticity, and eventually facilitates the establishment of a stable drug-resistant phenotype. Several practical challenges remain, including complexity in epigenetic features, dynamic epigenome changes, and interaction between epigenetic features. Specifically, there are five major classes of epigenetic markers: methylation, acetylation, ubiquitination, SUMOylation, and phosphorylation, which can interact with thousands of locations in the genome [22]. Furthermore, although single cell sequencing techniques can provide single cell resolutions of epigenome changes within a tumor, it provides only a static view and thus cannot provide information on the structural and epigenetic changes that cells may go through to acquire drug resistance.

Here, we combined in situ epigenetic tracking tools with data-driven image analysis to understand how DNA methylation and selected histone modification change under drug selection pressure. Our results indicate that drug resistant cells possess a significantly different epigenetic profile in comparison to regular cancer cells, which is highlighted by increases in repressive epigenetic markers and decreases in activation markers. These groups were well characterized by their epigenetic features and a data-driven clustering approach was able to identify significantly different features between the populations. We also observed that the treatment of cancer cells with tamoxifen revealed a significant increase in DNA methylation but not histone trimethylation in the surviving cell population. MCF7 cells exhibited a distinctive distribution of DNA methylation after treatments, indicating that plastic adaptation through DNA methylation is likely the key to resistance acquisition.

## 2. Materials and Methods

### 2.1. Culture of MCF7 and MCF7-Tamoxifen-Resistant Cell Line

MCF7 cells were cultured in a DMEM medium (Gibco, Waltham, MA, USA) supplemented with 10% FBS (Atlanta Biological, Flowery Branch, GA, USA) and 1% penicillin/streptomycin. MCF7 cells resistant to tamoxifen (TamR) were established by continuously culturing MCF7 with 10 μM tamoxifen for 3 weeks (such as 3-day on, followed by 2-day off treatments) similar to descriptions in the literature [23,24]. The established TamR cell line exhibited a different growth rate and resistance to tamoxifen, as summarized in Figure S1 (Supplementary Materials). TamR was then continuously cultured in a medium with 10 μM tamoxifen to maintain its resistant phenotype.

### 2.2. Proliferation Assay

Proliferation assay of MCF7 and TamR with and without tamoxifen was performed by measuring the cell growth in a 2D culture. Specifically, MCF7 and TamR cells were seeded at 30% confluency in a 96 well plate. Cells were imaged every 24 h using an IncuCyte S3 Live-cell Analysis System (Essen Bioscience, MI, USA) for 5 days. Relative cell numbers were quantified using cell coverage area.

### 2.3. Invasiveness Assay

The cell invasiveness assay was performed using a Transwell insert (Corning, NY, USA) following an established protocol [25,26]. Specifically, MCF7 or TamR cells were seeded on the top of a Transwell membrane. After cell attachment, TamR cells were cultured in a medium with 10 µM tamoxifen, and MCF7 cells were cultured in a medium without tamoxifen. After 72 h, the cells were fixed by immersing the Transwell membrane in a fresh 4% paraformaldehyde solution. The cell nuclei were then stained in a 5 µg/mL DAPI solution followed by imaging of both sides of membranes via a fluorescence microscope to determine the numbers of non-invasive and invasive cells, respectively.

### 2.4. Immuno-Staining

All of the cells were seeded into a half-area 96-well imaging plate (Corning, New York, NY, USA). Immunostaining was performed following established protocols [27,28]. Specifically, cells were fixed with a 4% paraformaldehyde solution for 15 min, and permeabilized overnight with 1% TritionX-100 in PBS before blocking using 3% BSA and 0.5% TritonX-100 in PBS. An additional denaturation step using 4N HCl followed by neutralization with 100 mM Tris HCl (pH = 8.0) was included for 5mC staining before blocking. The cells were then stained for 5mC, H3K9me3, H3K27me3, and H3K27ac using primary antibodies anti-5mC (61479, Active Motif, Carlsbad, CA, USA), anti-H3K9me3 (ab8898, Abcam, Cambridge, MA, USA), anti-H3K27me3 (ab192985, Abcam, Cambridge, MA, USA), and anti-H3K27ac (ab45173, Abcam, Cambridge, MA, USA), respectively. The cells were also stained for Ki67 (PA5-16785, Invitrogen, Waltham, MA, USA), a commonly used proliferation marker [29,30,31]. The cells were then washed in PBS three times before being stained for a secondary antibody, including Goat-anti-mouse coupled with Alexa 488 (A-11001, Invitrogen, Waltham, MA, USA) for 5mC, and Goat-anti-rabbit coupled with Alexa 568 (ab175471, Abcam, Cambridge, MA, USA) for histone modifications and Ki67. After that, the cells were washed in PBS, stained for nucleus using Draq 5 (62251, Invitrogen, U.S.), and then imaged using a fluorescent microscope.

### 2.5. Live Cell Exposure

MCF7 cells were plated onto a µ-Slide 8 well slide (Ibidi). The cells were transfected with live cell probes for ^me^CpG [32] or H3K9me3 [33] (see Table S1 (Supplementary Materials) for detailed sequence) using lipofectamine 3000 and P3000 reagent (L3000015, ThermoFisher Scientific) at ~50% confluency. An mEGFP plasmid, as we described in our prior work [34], was also transfected as an expression control. The cells were imaged and treated with 10 µM tamoxifen 24 h after transfection.

### 2.6. Fluorescent Microscopy

Confocal microscope images were collected using a Nikon Eclipse Ti-2 inverted microscope equipped with 488, 532, and 640 nm laser lines and a 60 ×/1.40 NA oil objective. All confocal images were collected at a thickness of 1 μm. 2D z-stack projections of the cells were obtained via the z-projection module of ImageJ.

### 2.7. Data Analysis and Statistics

All data were reported as mean ± SD, each containing more than three independent replicates unless otherwise stated in the paper. All data processing were performed using RStudio. Principle component analysis (PCA) was performed using the prcomp function in R. Clustering of PCA results was performed using the k-means clustering method using the kmeans function in R. The figures and part of the statistical analysis were performed using OriginPro (2019b).

## 3. Results

### 3.1. Characterize Phenotypic Changes between MCF7 and TamR

TamR cells, tamoxifen resistant MCF7 cells, were cultured by continuously growing MCF7 in 10 μM Tam. We performed a phenotypic assessment of TamR cells, as shown in Figure S1 (Supplementary Materials). The addition of Tam significantly decreased the growth rate of MCF7 cells, as shown in Figure S1A (Supplementary Materials). The doubling time of MCF7 increased from 2.85 ± 0.49 days to 5.02 ± 0.82 days in 10 µM Tam, as expected. The growth rate of TamR cells was significantly higher than that of MCF7 cells in a medium with 10 μM of Tam. To further confirm the observed difference in growth rates, we stained cells using Ki67, a cellular proliferation marker, to determine the percentage of proliferating cells after 48 h of Tam treatment. Typical staining images are shown in Figure S1B (Supplementary Materials). Moreover, ~40% of MCF7 cells were found to be Ki67 positive in a Tam-free culture medium. This percentage was significantly decreased with increasing the Tam concentrations for MCF7 cells, specifically ~10% and 5% for 5 and 10 μM of Tam, respectively. The percentage of Ki67 positive cells, however, remained unchanged for TamR, independent of Tam concentrations. These results confirmed that our TamR cells exhibited different growth phenotypes in response to Tam treatment compared to MCF7. An invasiveness assay was carried out using a Transwell insert, with the results shown in Figure S1C (Supplementary Materials). The percentage of cells that migrated from one side of the membrane to the other side was quantified using DAPI nucleus staining. Furthermore, ~16% TamR cells migrated through the membrane, while a minimal amount (<1%) of MCF7 cells migrated, suggesting TamR is more invasive than MCF7. The cultured TamR thus exhibited the expected phenotypical behavior of tamoxifen resistant breast cancer cell lines, consistent with the literature observations [35,36].

We then proceeded to evaluate the difference in cellular morphology between MCF7 and TamR. Typical Draq5 stained cell nuclei colocalized with their corresponding DIC images were summarized in Figure S2A (Supplementary Materials). The nuclear area and eccentricity were determined using an identified cell nucleus via a customized CellProfiler 3.1.8 (Broad Institute) pipeline [37]. Compared to MCF7, TamR cells exhibited a significantly larger nuclear area (~22% increase in population mean). Although the range of the nuclear area was similar between MCF7 and TamR, a significantly greater fraction of larger cells existed in TamR, as shown in Figure S2B (Supplementary Materials). A lower mean nuclear eccentricity was observed for TamR cells. TamR cells displayed a larger range of eccentricity compared to the MCF7 cells and had a broader distribution. These results collectively suggested that there were significant morphological differences between MCF7 and TamR cells, whereas the MCF7 cells exhibited a relatively more uniform and elliptical shape.

### 3.2. Assess Epigenome Difference between MCF7 and TamR

**Figure 1:**
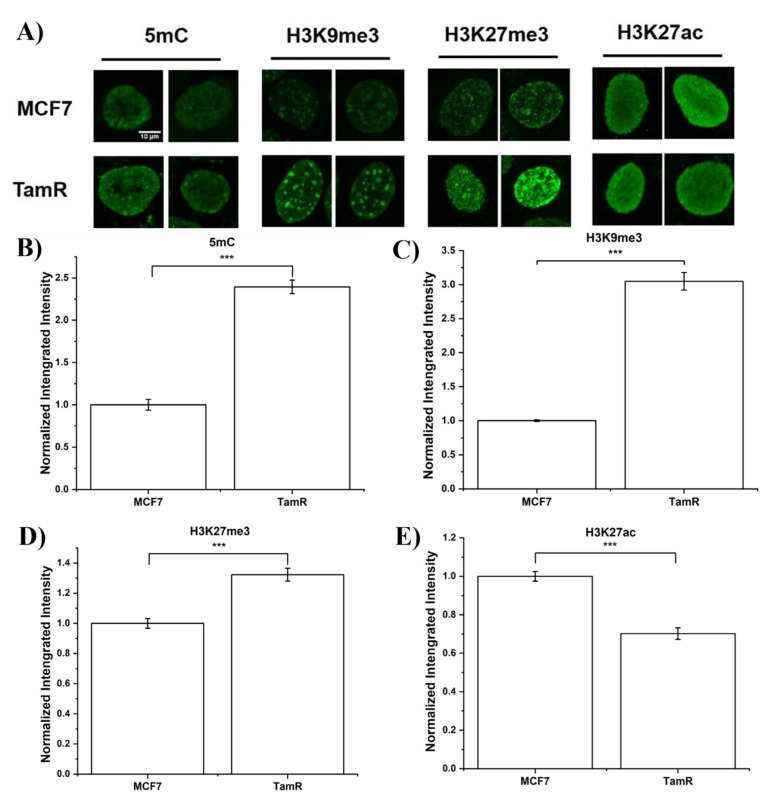
(**A**) Representative images of MCF7 and TamR cells stained with 5mC, H3K9me3, H3K27me3, and H3K27Ac antibodies. Scale bar = 10 µm. (**B**–**E**) Bar plot of nuclei integrated intensity of the two cell lines (MCF7 and TamR) stained with marker (**B**) 5mC, (**C**) H3K9me3, (**D**) H3K27me3, and (**E**) H3K27Ac. Error bar represents standard error. *N* > 100 cells in each group. *** indicates statistical difference with *p* < 0.001 calculated by a one-way ANOVA analysis followed by Tukey’s post-hoc test.

MCF7 and TamR were individually stained for 5mC, H3K9me3, H3K27me3, and H3K27ac, with typical staining images as shown in Figure 1A and Figure S3A–D (Supplementary Materials). These markers were selected because of their significance in modulating the gene expression [38,39,40,41], cell reprogramming [42,43], and cancer progression [44,45,46]. Integrated intensity per nuclei is commonly used to quantify changes in enzyme levels [47], and thus was used here to determine the changes in epigenetic modification levels within the population. Instead of comparing only the averaged intensity, we evaluated the distribution of integrated intensity of ~ 400 cells from three independent replicates for each epigenetic marker to highlight heterogeneity within the cell populations.

MCF7 and TamR cells stained for 5mC, H3K9me3, and H3K27me3 exhibited distinctive foci-like structures within the nucleus, representing heterochromatin regions. H3K27ac staining displayed a diffusive pattern, as expected. TamR cells exhibited a higher intensity for 5mC staining, resulting in a mean that was increased by 2.4 folds when compared to the MCF7 cells (see Figure 1B). In addition, TamR showed a broader distribution with a tail at the high-intensity bins, as shown in Figure S4A (Supplementary Materials). This is consistent with the previous reports that Tam resistant MCF7 cells exhibit a higher global DNA methylation level [48,49]. When examining H3K9me3, another repressive marker of the constitutive heterochromatin, we observed a similar pattern of increase in mean, ~3 folds in this case (see Figure 1C). Again, TamR cells showed a broader distribution of integrated intensity enriched with a higher intensity subpopulation compared to MCF7 cells (see Figure S4B (Supplementary Materials)). The staining of another repressive marker, H3K27me3, a facultative heterochromatin marker, showed a ~30% increase in integrated intensity from MCF7 to TamR. Unlike 5mC and H3K9me3, the H3K27me3 staining pattern showed a shift in distribution from a low to high intensity of TamR compared to MCF7, while the width of the distribution remained almost the same (see Figure 1D and Figure S4C (Supplementary Materials)). This is consistent with previous findings that Tam resistant breast cancer showed a higher expression of EZH2, a H3K27 methyltransferase, compared to the Tam responsive ones [50]. We also stained for an activation marker, H3K27ac. There was a ~30% decrease of integrated intensity in TamR compared to MCF7, as shown in Figure 1E. TamR showed a skewed distribution enriched with cells of low H3K27ac levels compared to MCF7 (Figure S4D (Supplementary Materials)). The expression of estrogen receptor (ER) is H3K27ac dependent [51]. The ER signaling pathway is inhibited by H3K27 acetyltransferase inhibitors [52], indicating that our observed H3K27ac decrease may have contributed to the downregulation of ER signaling and subsequently conferred Tam resistance. Overall, we observed a global increase in the gene silencing marker in TamR cells, including 5mC, H3K9me3, and H3K27me3, and a decrease in gene activation marker, H3K27ac.

**Figure 2:**
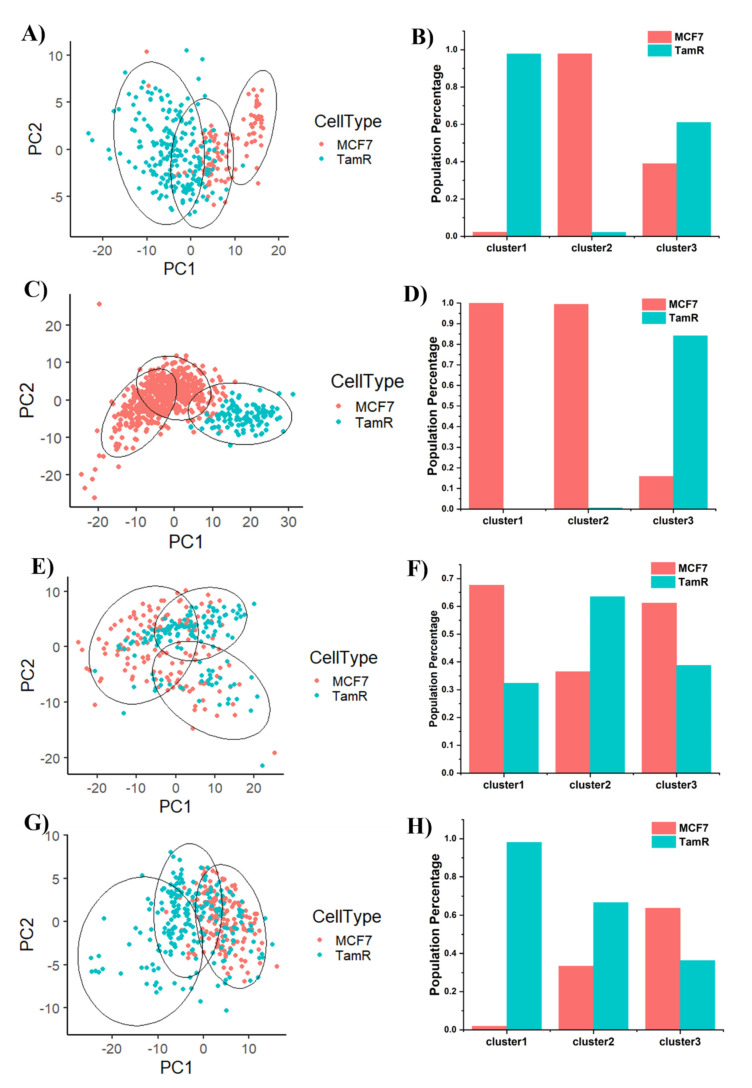
Principle component analysis (PCA) of MCF7 and TamR cells stained with (**A**) 5mC, (**C**) H3K9me3, (**E**) H3K27me3, and (**G**) H3K27ac. Circles represent k-means clustering results. Percentage of MCF7 and TamR cells in each cluster are calculated and plotted in (**B**) 5mC, (**D**) H3K9me3, (**F**) H3K27me3, and (**H**) H3K27ac.

Integrated intensity changes are typically associated with the global abundance of epigenetic modifications. The spatial distribution of selected epigenetic markers reflects chromatin compactness, 3D genome organization, and thus carries crucial information about gene regulation as extensively reviewed in literature [53,54,55,56]. Other than histone acetylation (H3K27ac) that exhibits a diffusive pattern within the nucleus, other selected repressive markers, 5mC, H3K9me3 and H3K27me3, all reveal foci and island-like features. We thus characterized these sub-nuclear features using CellProfiler. Typical feature identification for each type of staining is shown in Figure S5 (Supplementary Materials). To further elucidate the heterogeneous distribution and better visualize multi-dimensional measurements including intensity and spatial organization of selected markers, principle component analysis (PCA) of MCF7 and TamR cell lines was performed. We started by including intensity, texture and morphology related features of each epigenetic marker measured from immunofluorescent images. Every single cell is mapped back to the PC space as a single dot as shown in Figure 2 To further characterize possible subpopulations distinguished by selected epigenetic markers, we performed k-means clustering as circled out on PCA plot. Elbow plots were used to determine the number of clusters (Figure S6 (Supplementary Materials)). Identified clusters are circled out in the PCA plots (see Figure 2). The percentage of MCF7 or TamR cells in each cluster are determined and plotted in Figure 2B,D,F,H. We also performed a Lasso regression analysis to determine the top features used to distinguish between two cell lines as shown in Table S2 (Supplementary Materials).

TamR and MCF7 cells stained with 5mC show distinct separation from two cell types, as shown in Figure 2A. Specifically, there are two subpopulations of MCF7, one of which overlaps with TamR and the other one is distinct from TamR. This indicates that DNA methylation patterns of MCF7 cells are heterogeneous with a subpopulation that shares similar DNA methylation features with TamR. Our observation is further confirmed by bar plot in Figure 2B, with cluster 1 dominated by TamR, cluster 2 dominated by MCF7 and cluster 3 as a mixture of both. We then examined the top features with the highest Lasso coefficient (Table S2 (Supplementary Materials)). The features that distinguish TamR and MCF7 are intensity and foci texture-based features, indicating global 5mC level and heterochromatin organization play an essential role for drug resistance development. For H3K9me3, MCF7 and TamR are distinctively separated with minimal overlap (Figure 2C). Cluster 1 and 2 are 100% MCF7 cells. Cluster 3 is dominated by TamR with 80% (Figure 2D). H3K9me3 PCA result indicates that TamR forms a new population compared to MCF7. Lasso analysis shows that the best features that distinguish two populations are all texture-based features, indicating instead of global abundance, H3K9me3 distribution inside the nucleus exhibits more significant changes during resistance acquisition. Another heterochromatin marker, H3K27me3 shows less separation with a large amount of overlap of MCF7 and TamR on PC space (Figure 2E), which is further confirmed by uniform distribution in 3 clusters (Figure 2F). This result indicates that H3K27me3 global abundance and distribution are not sufficient to distinguish MCF7 and TamR. We then examined acetylation marker H3K27ac and plotted it on PC space (Figure 2G). MCF7 shows a narrower distribution compared to TamR. Half of TamR cells demonstrate similar patterns as MCF7 (clusters 2 and 3) while the rest half of TamR forms a unique subpopulation, noted as cluster1 (Figure 2H). From Lasso analysis, texture-based features showed a higher Lasso coefficient compared to intensity-based features (Table S2 (Supplementary Materials)), suggesting acetylation marker organization distinguishes MCF7 and TamR better compared to global abundance. Overall, 5mC and H3K9me3 show the most distinctive clustering of MCF7 and TamR cells and thus warrant further investigation.

### 3.3. Tracking Epigenome Changes during Tam Treatment

**Figure 3:**
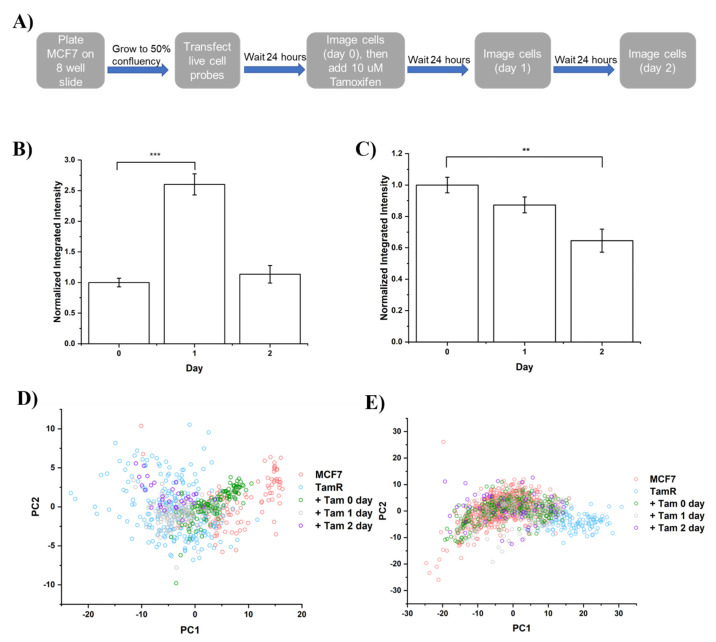
(**A**) Schematic illustration of live cell tracking imaging and treatment timeline. (**B**,**C**) Barplot of normalized integrated intensity of single cells transfected with (**B**) dMBD1 probe targeting ^me^CpG and (**C**) H3K9me3 probes. Normalization was performed by dividing the intensity of the TAM treated group by the intensity of the non-treated group. (*N* > 50 cells. **: *p* < 0.01; ***: *p* < 0.001 (one-way ANOVA followed by Tukey’s post-hoc test). (**D**,**E**) PCA of MCF7 cells transfected with a (**D**) ^me^CpG probe or (**E**) H3K9me3 probe mapped to PC space constructed from 5mC or H3K9me3 immunostaining.

From PCA results, we observed distinctive separation of MCF7 and TamR from staining of 5mC and H3K9me3. However, the dynamic transition of selected epigenetic markers during resistance acquisition remains unknown. Previously, our group engineered in-situ sensors to track epigenome changes, with comparable spatial resolution and accuracy to immunostaining [32,33,34,57]. Briefly, these probes were engineered using native epigenetic reader domains that minimally perturbed cellular function, fused with fluorescence protein with detailed probe sequences, as shown in Table S1 (Supporting Information). MCF7 cells were imaged every 24 h following a treatment/imaging schedule, as outlined in Figure 3A.

We first analyzed the changes in the integrated nuclei intensity, representing how the epigenetic marker responds to Tam treatment on a global level. We observed a dramatic increase (~2.5 fold) in the global 5mC level after 1-day of treatment of Tam, as shown in Figure 3B. The 5mC level went back to the equivalent level with no treatment group after 2 days of Tam treatment. This result suggests that DNA methylation showed an acute increase in response to Tam treatment, while it restored its homeostasis in response to long term treatment. H3K9me3 showed no significant change in integrated intensity after 1-day of treatment but showed a ~35% decrease after 2 days of treatment, as shown in Figure 3C.

To further investigate the resistance development trajectory, we analyzed live cell probe tracking data with CellProfiler and mapped them to the PC space constructed with the immunostaining data of MCF7 and TamR. From the PCA plot of the tracking data of the DNA methylation pattern (Figure 3D), we observed a population shift from MCF7 towards TamR with increasing the time of treatment. Before treatment, the cells were in a subpopulation where MCF7 and TamR overlap. After 1 day of treatment, cells shifted towards the TamR exclusive subpopulation with a moderate overlapping with the treatment naïve group. After 2 days of treatment, the cells shifted further towards the TamR exclusive subpopulation with minimal overlap with before the treatment group. Our data suggest a gradual shift of the 5mC pattern towards a resistant phenotype with prolonged Tam treatment.

PCA of H3K9me3 tracking shows a different pattern (Figure 3E). As expected, transfected cells overlapped with MCF7 cells before Tam treatment. There was no obvious population shift after 1 day of treatment of Tam. After 2 days of Tam treatment, H3K9me3 showed a slightly broadened distribution, but still within the MCF7 cluster, suggesting that 2 days of Tam treatment was not sufficient to trigger the development of a drug resistant cell phenotype.

## 4. Discussions

### 4.1. Epigenome and Morphological Changes Correlate with Drug Resistance in a Breast Cancer Cell Line

Nuclear morphology has been discovered to be correlated with cancer metastatic potential and progression state. For example, an increased nucleus area was observed in a highly metastatic osteosarcoma cell line [58]. An altered nuclear morphology is becoming one of the defining factors in cancer quantification, due to how this alteration can affect chromatin organization and function [59,60]. A condensed and fragmented cell nuclei was observed in tamoxifen-coated nanoparticle treated MCF7 cells [61]. The Tam-resistant cells that we used also exhibited a larger nuclear size and decreased eccentricity, which may have affected the chromatin dynamics of the nucleus.

The acquisition of tamoxifen resistance in the MCF7 breast cancer cell line had a clear effect on several major epigenetic markers, particularly DNA methylation and H3K9me3. However, we observed changes in H3K27me3 and H3K27ac as well. Global DNA hypermethylation has been widely found in chemotherapy resistant breast cancer models, which is consistent with our observation in MCF7 and TamR cell lines. An 85% increase in DNA methylation was found in docetaxel resistance MCF7 cell line compared to the docetaxel sensitive cell line [62]. Methylation analysis from a patient-derived xenograft of a triple-negative breast cancer model revealed hypermethylation on the promotor region and CpG islands for docetaxel resistance samples. The methylation of signature CpGs identified in this report could potentially predict treatment outcome [63]. Hypermethylation was also observed in trastuzumab resistant HER2-positive breast cancer [64]. Mechanistically, there is evidence showing that chemotherapy drugs promote ER binding to the promoter region of DNMT1, thus resulting in the hypermethylation of drug resistant MCF7 cells [65]. Similarly, H3K9me3 accumulation has been seen in drug resistance of several cell lines, including breast, colon, and lung [66]. Coupled with 5mC, H3K9me3 has been recognized as heterochromatin markers [67]. Consistent with the increased abundance of heterochromatin markers, an increase of another repression marker, H3K27me3, was also observed in TamR. This is consistent with the previous finding that Tam resistant breast cancer shows a higher expression of EZH2, a H3K27 methyltransferase, compared to Tam responsive ones [50]. The H3K27me3 level has also been postulated to be associated with cancer stem cell populations. The expression level of H3K27me3 demethylase, KDM6A, is reduced in a stem-like population of breast cancer cell lines [68]. Another cancer stemness promoting gene, HOXA5, was also found to be regulated by H3K27me3 in the tamoxifen resistant MCF7 cell line [24]. Therefore, it is not surprising that the drug resistant population has an elevated level of H3K27me3. The inverse relationship between the repressive and activation markers strongly indicates that the development of drug resistance is driven by the silencing of genes. It is possible that this silencing is directed toward apoptosis genes, as Tam is known to cause apoptotic and antiproliferation gene activation [69,70]. The PCA plots of 5mC, H3K9me3, and H3K27ac display significant divergences between the populations, indicating that the drug resistant cells have acquired a different epigenetic state than the MCF7 cells. As epigenetic state is heritable [71], it is possible that the TamR cells are passing this epigenetic state to their daughter cells and accelerating the rate of drug resistant tumor development. Divergence from PCA plot also suggests that 5mC and H3K9me3 global abundance and spatial organization are potential biomarkers for the drug resistant state of breast cancer.

### 4.2. Dynamic Reprogramming of Epigenome during Tam Treatment

Assessment tools such as RNA-seq and immunostaining are useful endpoint assessments to determine epigenetic contributions to drug resistance. They, however, lack the ability to generate data for a multitude of time points due to their cost and labor intensity. Our live cell probes offer us unique advantages in monitoring epigenetic changes in real-time to understand early epigenetic changes as discussed above. To understand how the development of drug resistance begins we treated MCF7 cells with Tam and captured the immediate changes in the epigenetics of 5mC and H3K9me3 using our live cell probe. Cells transfected with the live cell probes showed significant cell death after treatment with Tam. The surviving cells exhibited significant increases in DNA methylation immediately after treatment but decreases in H3K9me3. After mapping live cell data to immunostaining, we observed a gradual shift of the DNA methylation pattern from a MCF7-like subtype to a TamR-like subtype. Our data suggest that DNA methylation of the surviving cells went through adaptive changes in response to Tam treatment. After 2 days of Tam treatment, the DNA methylation pattern evolved as a distinctive new population compared to that before treatment. This result suggests that instead of selection toward a pre-existing DNA methylation pattern that favors cell survival, the cells adapted themselves by rewiring DNA methylation patterns in response to Tam treatment. We did not observe a similar trend in H3K9me3. Different from DNA methylation patterns that went through dramatic changes immediately after treatment, H3K9me3 may play a passenger role by passively rewiring heterochromatin structure following DNA methylation changes. From here, we conclude that instead of passive selection, rapid adaptation of DNA methylation may be a potential driver in acquisition of tamoxifen resistance, therefore leading to heterochromatin rewiring and later changes in other heterochromatin markers.

## 5. Conclusions

The treatment of drug resistant cancers remains one of the most critical issues in modern medicine. The mechanisms of drug resistance however remain elusive. The intrinsic heterogeneity of cancer cells, also known as tumor heterogeneity, can partially contribute to drug resistance, necessitating single-cell-based analysis. In this work, we examined morphological and epigenetic changes in Tam responsive and resistant MCF cells. An examination of the epigenetics of these cell lines through immunostaining shows that TamR cells exhibit higher levels of DNA methylation, H3K9me3, and H3K27me3, as well as lower levels of H3K27ac. A data driven PCA analysis revealed distinct clusters based on drug responsiveness, with the largest distinction observed from 5mC and H3K9me3 markers, suggesting 5mC and H3K9me3 as potential biomarkers for tamoxifen resistant breast cancer cells. Furthermore, we used live cell compatible probes to track 5mC and H3K9me3 changes immediately after Tam treatment. We found that the surviving population could rapidly increase DNA methylation after treatment. PCA further confirms that MCF7 cells adaptively change DNA methylation towards a pattern more like TamR. Changes in H3K9me3 are less distinctive and slower. Collectively, our results indicate that DNA methylation plays a prevailing role in tamoxifen resistance acquisition, while H3K9me3 plays a passenger role in resistance development. Our analysis based on the foci-pattern also suggests that heterochromatin organization may act as a potential driver for the acquisition of tamoxifen resistance in MCF7.

## Data Availability

Data available upon request.

## Supplementary Materials

The following supporting information can be downloaded at: https://www.mdpi.com/article/10.3390/life12030438/s1. Table S1: Amino acid sequences of in situ epigenetic probes; Table S2: Top ranked features contributing to the distinction between MCF7 and TamR cells with their respective Lasso coefficients; Figure S1: Growth rate, proliferation and invasiveness of MCF7 and TamR cells; Figure S2: Nucleus staining and nuclear morphology of MCF7 and TamR cells; Figure S3: Representative images of MCF7 and TamR cells stained with 5mC, H3K9me3, H3K27me3 and H3K27ac antibodies; Figure S4: Histograms of integrated intensity of 5mC, H3K9me3, H3K27me3 and H3K27ac from single nuclei of MCF7 and TamR cells; Figure S5: Representative images of nucleus and foci identification using CellProfiler; Figure S6: Elbow plot for determining cluster numbers.
