# Supplementary material for: Single-Cell Image-Based Analysis Reveals Chromatin Changes during the Acquisition of Tamoxifen Drug Resistance
Source: Life (Basel). 2022 Mar 17;12(3):438. doi: 10.3390/life12030438 (PMC8950147; doi:10.3390/life12030438)
Supplement: Supplementary file 1 [file life-12-00438-s001.zip › life-1628334-supplementary.pdf]

life

Supporting Information

Single-Cell Image-Based Analysis Reveals Chromatin Changes during the Acquisition of Tamoxifen Drug Resistance

Han Zhao [1], Li F. Lin [1], Joshua Hahn [1], Junkai Xie [1], Harvey F. Holman [1] and Chongli Yuan [1,2]

[1] Davidson School of Chemical Engineering, Purdue University, West Lafayette, IN 47907, USA
[2] Purdue University Center for Cancer Research, Purdue University, West Lafayette, IN 47906, USA

1. **SUPPORTING TABLES**
2. **SUPPORTING FIGURES**

1. SUPPORTING TABLES:

Table S1. Amino acid sequences of *in situ* epigenetic probes

Probe	Amino acid sequence*
meCpG Probe	10 20 30 40 50<br>**MPKKKRKV***GS GSGSGSGGS*E FAEDWLDSPA LGPGWKRREV FRKSGATAGR<br>60 70 80 90 100<br>SDTYYQSPTG DRIRSKVELT RYLGPAGDLT LFDFKQGIL*G GG*QFAEDWLD<br>110 120 130 140 150<br>SPALGPGWKR REVFRKSGAT AGRSDTYYQS PTGDRIRSKV ELTRYLGPAG<br>160 170 180 190 200<br>DLTLFDFKQG IL*GGG*QFVPV ATMVSKGEEL FTGVVPILVE LDGDVNGHKF<br>210 220 230 240 250<br>SVSGEGEGDA TYGKLTLKFI CTTGKLPVPW PTLVTTLTYG VQCFSRYPDH<br>260 270 280 290 300<br>MKQHDFFKSA MPEGYVQERT IFFKDDGNYK TRAEVKFEGD TLVNRIELKG<br>310 320 330 340 350<br>IDFKEDGNIL GHKLEYNYNS HNVYIMADKQ KNGIKVNFKI RHNIEDGSVQ<br>360 370 380 390 400<br>LADHYQQNTP IGDGPVLLPD NHYLSTQSKL SKDPNEKRDH MVLLEFVTAA<br>410 420<br>GITLGMDELY K*
H3K9me3 Probe	10 20 30 40 50<br>**MPKKKRKV***GS GSGSGSGGS*E FASQEFEVEA IVDKRQDKNG NTQYLVRWKG<br>60 70 80 90 100<br>YDKQDDTWEP EQHLMNCEKC VHDFNRRQTE KQKKLTWTTT SRIFSNNA*GS*<br>110 120 130 140 150<br>*GGGGGS*QFAS QEFEVEAIVD KRQDKNGNTQ YLVRWKGYDK QDDTWEPEQH<br>160 170 180 190 200<br>LMNCEKCVHD FNRRQTEKQK KLTWTTTSRI FSNNA*GSGGG GGS*QL*GSGG*V<br>210 220 230 240 250<br>DSCLPVATMV SKGEELFTGV VPILVELDGD VNGHKFSVSG EGEGDATYGK<br>260 270 280 290 300<br>LTLKFICTTG KLPVPWPTLV TTLTYGVQCF SRYPDHMKQH DFFKSAMPEG<br>310 320 330 340 350<br>YVQERTIFFK DDGNYKTRAE VKFEGDTLVN RIELKGIDFK EDGNILGHKL<br>360 370 380 390 400<br>EYNYNSHNVY IMADKQKNGI KVNFKIRHNI EDGSVQLADH YQQNTPIGDG<br>410 420 430 440 450<br>PVLLPDNHYL STQSKLSKDP NEKRDHMVLL EFVTAAGITL GMDELYK*

*: **Nucleus localization sequence**

Linker

Dimeric reader domains
GFP

Table S2. Top ranked features contributing to the distinction between MCF7 and TamR cells with their respective Lasso coefficients.

5mC Lasso Coefficient	Feature
0.285341	Intensity MaxIntensity
0.232295	Mean Foci Texture InfoMeas2
0.136416	Mean_Foci_Intensity_MinIntensity_
-0.11715	Mean_Foci_Texture_Contrast
-0.09716	Number of Foci_Count

H3K9me3 Lasso Coefficient	Feature
0.376078	Texture_Entropy
0.324195	Texture_SumAverage
-0.23239	Texture_DifferenceEntropy
-0.10407	Texture_InfoMeas2
0.090125	Texture_AngularSecondMoment

H3K27me3 Lasso Coefficient	Feature
-0.65099	Texture_DifferenceEntropy
-0.51921	Texture_AngularSecondMoment
0.337793	Intensity_LowerQuartileIntensity
0.113467	Intensity_IntegratedIntensity
-0.06518	AreaShape_Extent

H3K27ac Lasso Coefficient	Feature
-1.76596	Texture_Entropy
1.362131	Texture_SumEntropy
0.574568	Intensity_MedianIntensity
-0.29018	Intensity_MADIntensity
-0.20887	Intensity_MaxIntensity

2. SUPPORTING FIGURES

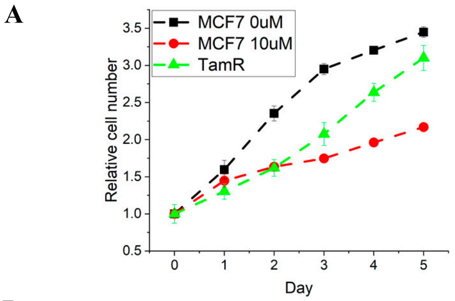

Cell line	Tamoxifen concentration (uM)	Doubling time (days)
MCF7	0	2.85 ± 0.49
MFC7	10	5.02 ± 0.82
TamR	10	3.02 ± 0.08

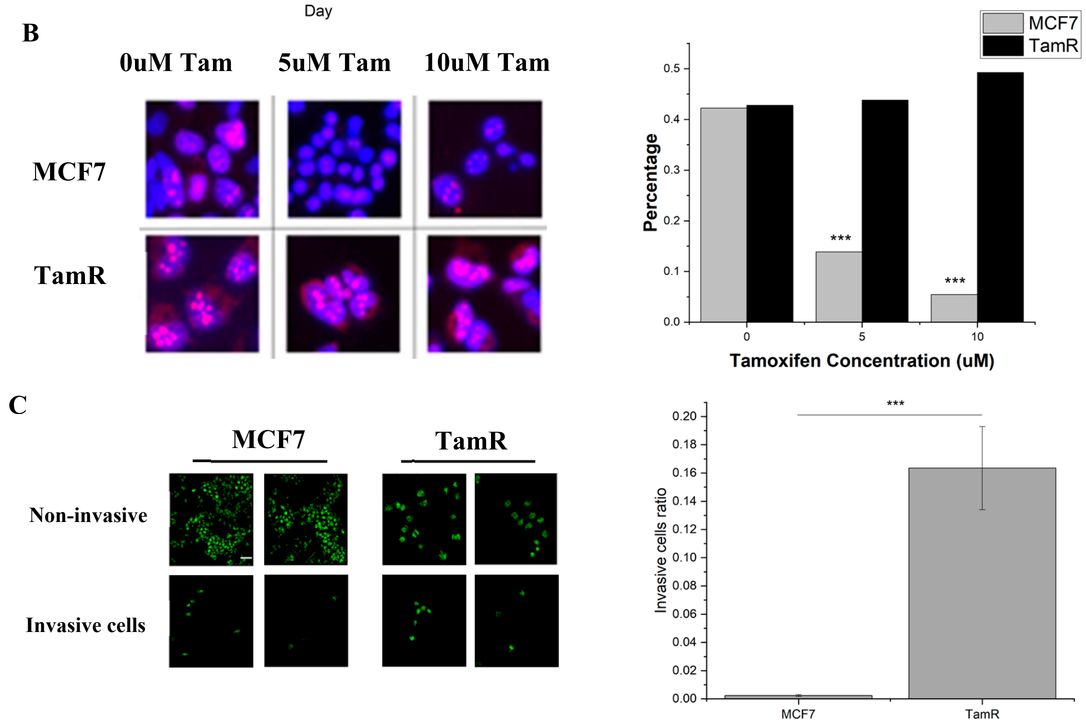

Figure S1. (**A**) Growth curve of MCF7 cells in the presence and absence of 10 uM TAM and TamR in the presence of 10 uM Tam and fitted doubling time. (**B**) Representative images of MCF7 and TamR cells stained with Ki67 and quantification of percentage of Ki67 positive cells. (**C**) Invasiveness assay of MCF7 and MCF7-R cell line with 0.4µm PET membrane. Scale bar = 20 µm. Cells remains on membrane surface are counted as non-invasive cells. Cells penetrate through the membrane are counted as invasive cells. Invasive cells divided by total cells number are used to calculate invasiveness ratio. Invasiveness assay was performed with n =3 biological replicates. *** : indicates $p < 0.001$.

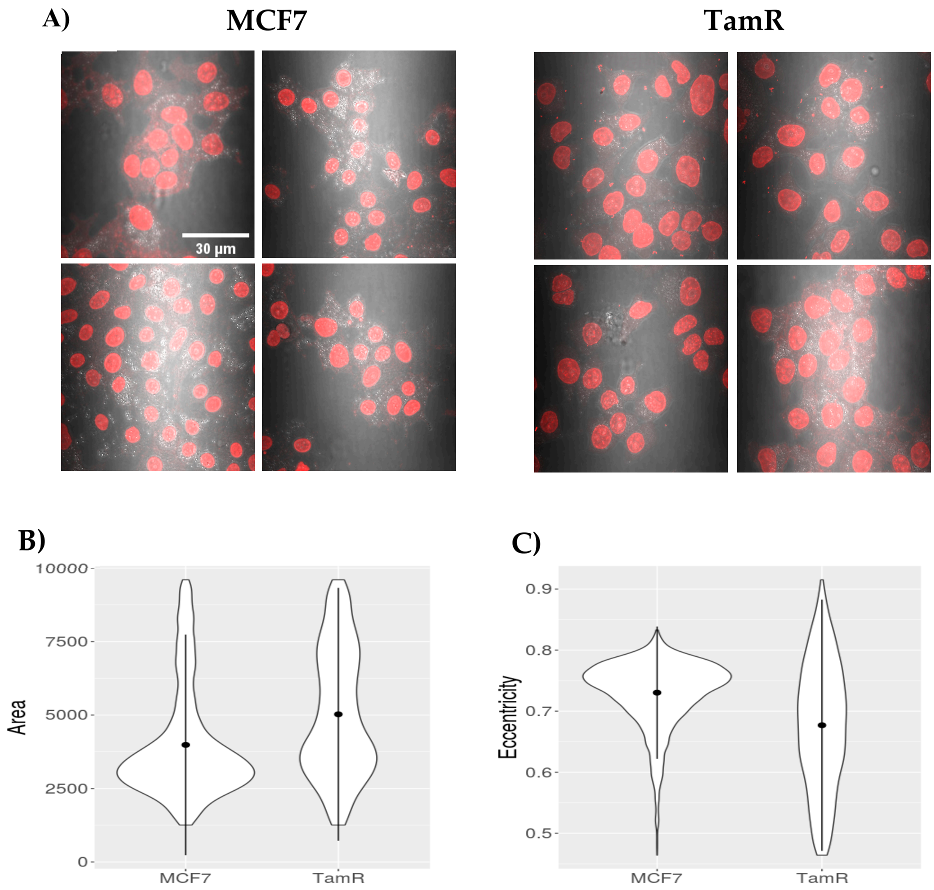

Figure S2. (**A**) Draq5 staining of MCF7 cells overlaid with DIC images (left), and Draq5 staining of TamR cells (right). (**B**) Violin plot of nuclear area in MCF7 and TamR cells. The dot represents the mean value and line represents standard deviation. (**C**) Eccentricity of MCF7 and TamR cell lines.

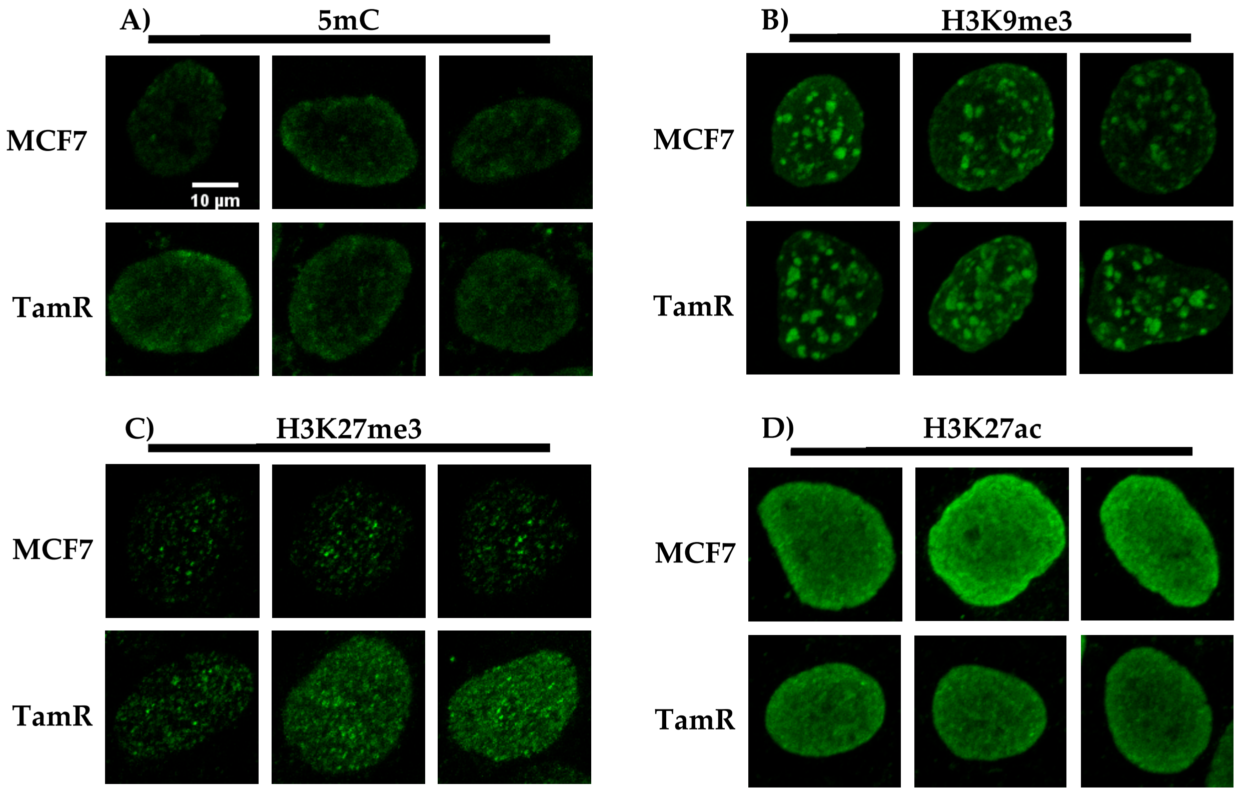

Figure S3. Representative images of MCF7 and TamR cells stained with (**A**) 5mC, (**B**) H3K9me3, (**C**) H3K27me3 and (**D**) H3K27ac antibodies.

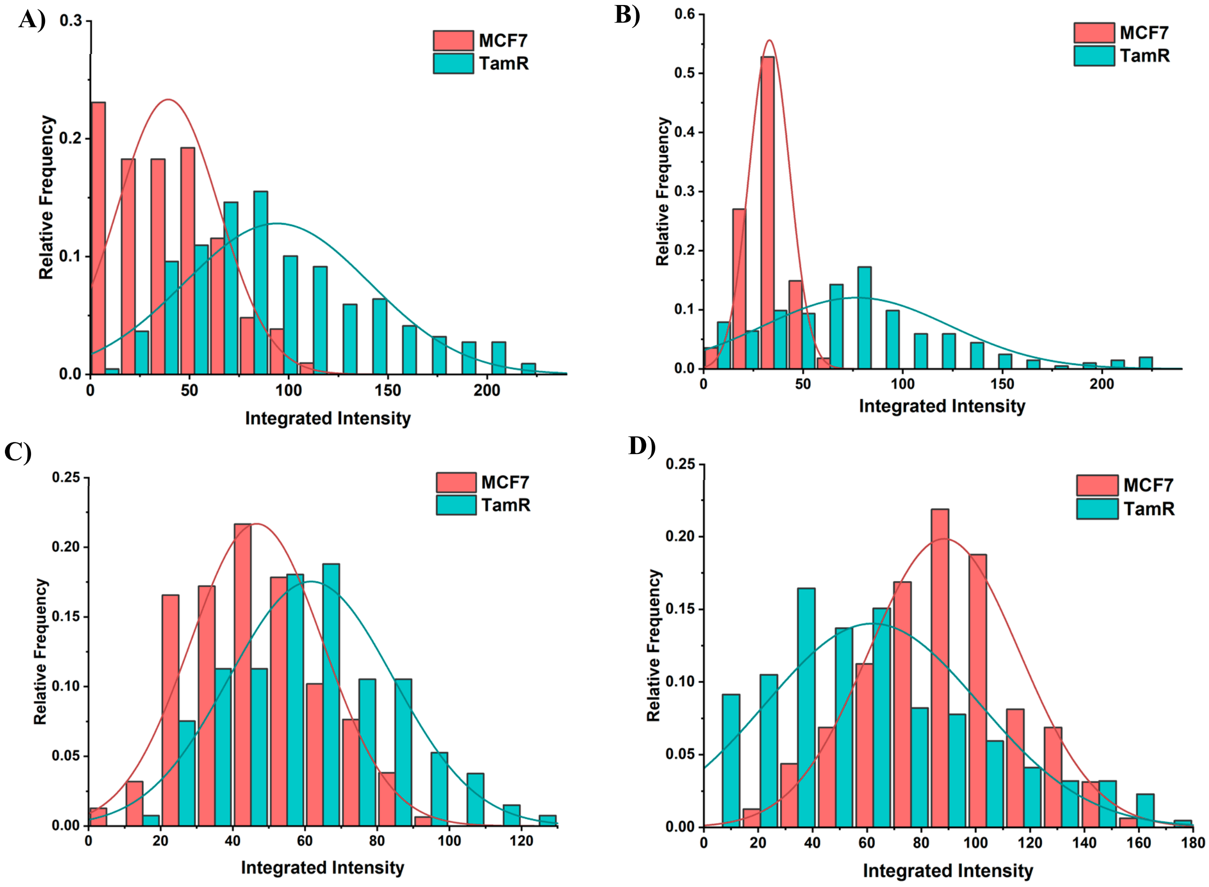

Figure S4. Histograms of integrated intensity of (**A**) 5mC, (**B**) H3K9me3, (**C**) H3K27me3 and (**D**) H3K27ac from single nuclei of MCF7 and TamR cells. All histograms are fitted with normal distribution.

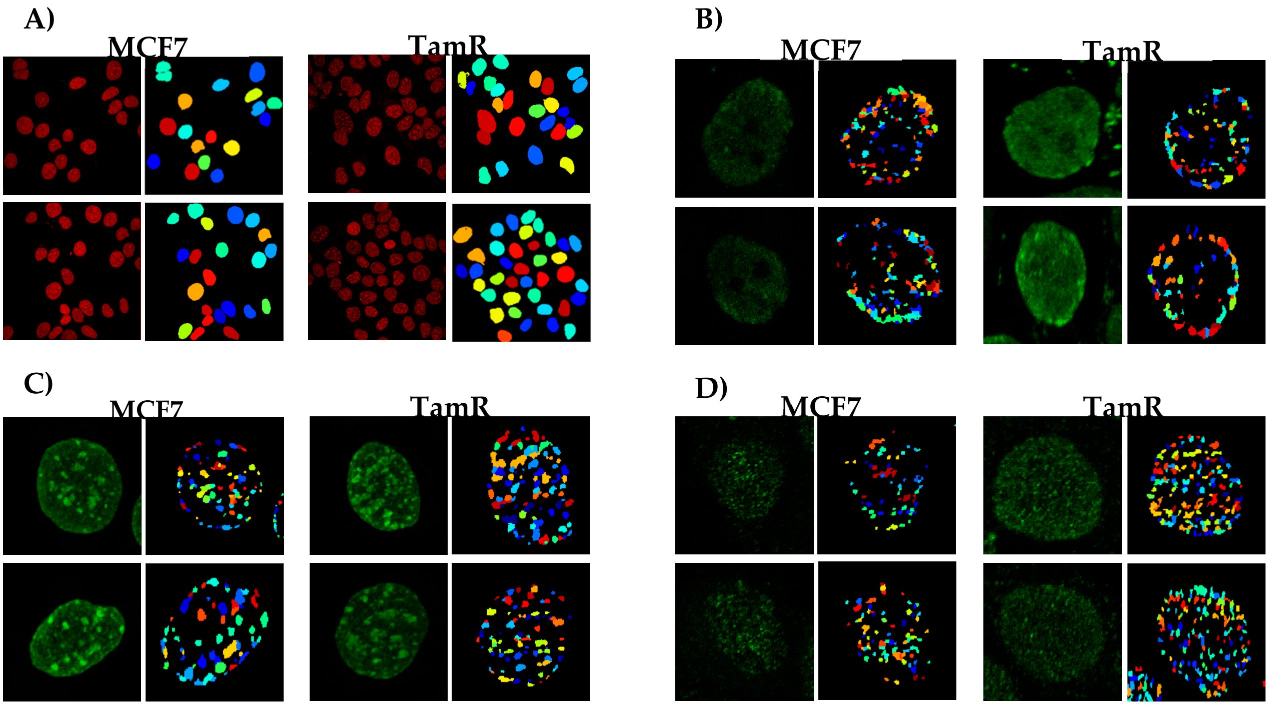

Figure S5. (**A**) Draq5 nuclear staining of MCF7 and TamR (red) and objects identified as nuclei by CellProfiler analysis (multicolor). (**B**) MCF7 and TamR cells stained for DNA methylation (green); DNA methylation islands representing foci-like features within nuclei identified by CellProfiler (multicolor). (**C**) MCF7 and TamR cells stained for H3K9me3 (green); H3K9me3 islands representing foci-like features within nuclei identified by CellProfiler (multicolor). (**D**) MCF7 and TamR cells stained for H3K27me3 (green); H3K27me3 islands representing foci-like features within nuclei identified by CellProfiler (multicolor).

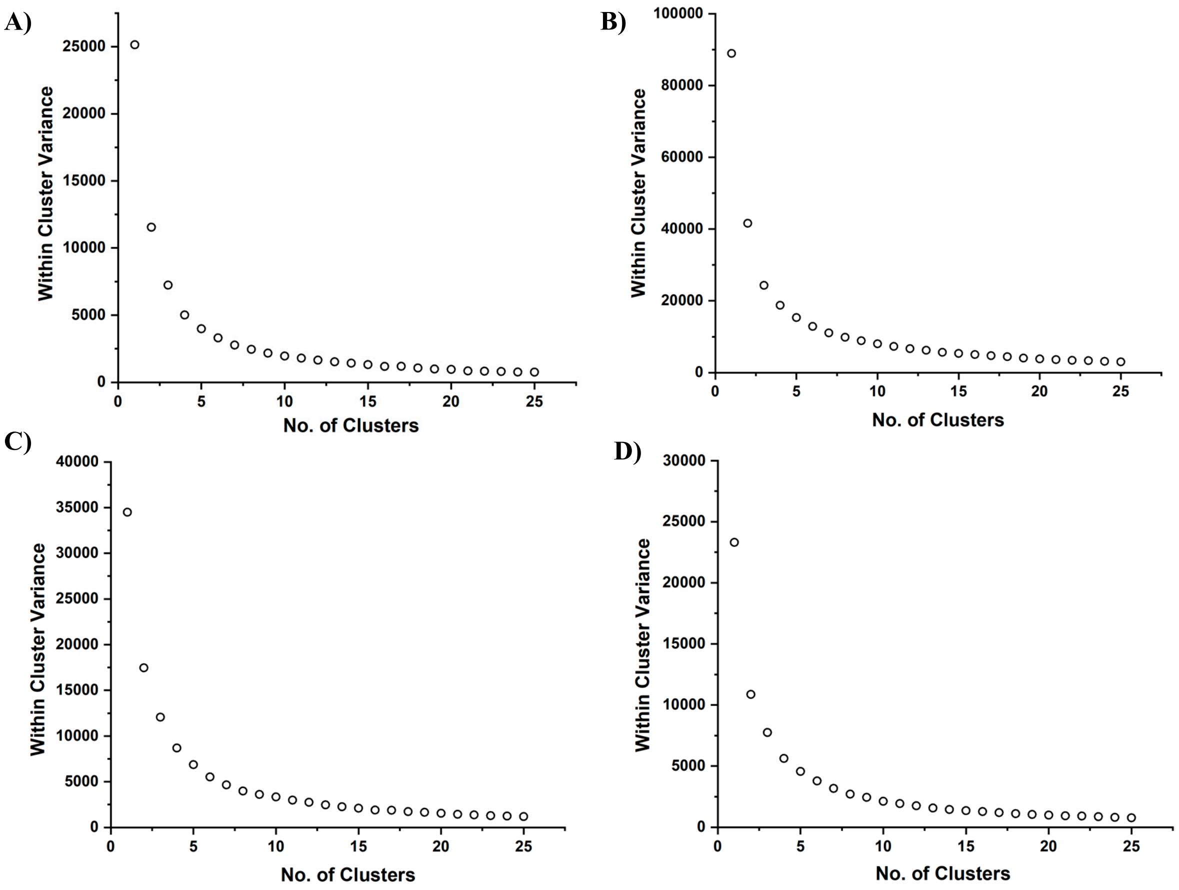

Figure S6. Elbow plot of attempted cluster numbers v.s. within cluster variance to determine number of clusters. (**A**) 5mC. (**B**) H3K9me3. (**C**) H3K27me3. (**D**) H3K27ac.
